# High Prevalence of Non-Vaccinated Oncogenic Human Papillomavirus Genotypes in High-Grade Squamous Intraepithelial Lesions of the Cervix: Thought-Provoking Results of a Detailed HPV Genotype Analysis

**DOI:** 10.3390/vaccines10050748

**Published:** 2022-05-10

**Authors:** Orsolya Rideg, Tímea Dergez, Kornélia Farkas, Krisztina Kovács, Endre Kálmán, Tamás Tornóczky, Angéla Oszter

**Affiliations:** 1 Department of Pathology, Medical School and Clinical Center, University of Pécs, 7624 Pécs, Hungary; ridegorsi@yahoo.com (O.R.); kovacs.krisztina2@pte.hu (K.K.); ke6100@gmail.com (E.K.); oszter.angela@pte.hu (A.O.); 2 Institute of Bioanalysis, Medical School and Clinical Center, University of Pécs, 7624 Pécs, Hungary; timea.dergez@aok.pte.hu (T.D.); nelli.farkas@aok.pte.hu (K.F.)

**Keywords:** HPV Direct Flow CHIP, HPV genotype profile, PAP smear, FFPE cone sample, HSIL, nonavalent HPV vaccine

## Abstract

Identification of HPV infection is usually performed on cytological specimens, despite the often transient virus types. HPV profile analysis of pathologically confirmed lesions can also be performed on formalin-fixed paraffin-embedded (FFPE) cone samples and should be taken as standard during follow-up. We compared HPV profiles of cytological and FFPE specimens of women diagnosed with HSIL. Archived PAP smears and FFPE cones from 49 patients were processed. For genotyping, the HPV Direct Flow CHIP test was used. All samples were positive. HPV profile agreement of the two sample types was 84.16–100%. Mono-infections occurred in 12.24% and 61.22% in PAP smears and FFPE specimens, respectively; while multi-infections were detected in 87.76% and 38.78%, respectively. The most abundant genotypes were HPVs 16, 31, and 51/33. Of all infections, 56.25% and 64.93% were caused by nonavalent vaccinated type (VT) HPVs; while 50.69% and 38.96% belonged to non-nonavalent VT HPVs, in PAP smears and FFPE specimens, respectively. Our results confirmed the importance of HPV genotyping of FFPE cone samples. We also confirmed a remarkable presence of non-vaccinated HPV types in HSIL cases indicating the importance of vaccine development.

## 1. Background

There are over 207 different human Papillomavirus (HPV) genotypes classified to date, by varying distribution and prevalence across different populations and geographical regions [1,2,3,4,5,6,7,8,9,10]. Approximately 40 of these HPVs are sexually transmitted to the anogenital region including about 15 so-called high-risk (HR) types, such as HPVs 16, 18, 31, 33, 35, 39, 45, 51, 52, 56, 58, 59, 68, 73, and 82, that have been classified as oncogenic and are found to cause anogenital cancers. Besides, the most frequent low-risk (LR) types associated with the anogenital region, are HPVs 6, 11, 40, 42, 43, 44, 54, 55, 61, 62, 67, 69, 70, 71, 72, 81, and 84 [11,12]. The majority of sexually active women will acquire transient HPV infection; most (>90%) cervical HPV infections are resolved by the host immune system within one or two years [13,14,15]. Cancer formation does not result from such infections, however, over time, if changes occur in either the viral genome, in the infected host cell, or both, transient infection becomes persistent [13,16,17]. Transient infection with multiple HPV genotypes is usually found in low-grade squamous intraepithelial lesions (LSIL), while the persistent, non-productive infection is usually presented in precancerous high-grade squamous intraepithelial lesions (HSIL), that frequently harbor a similar range of anogenital-infective HPVs compared to that found in invasive cervical cancer [1,13,18].

Although it is generally accepted that both HSIL and invasive cancers are mostly driven by a single HPV type, in about 60% of the cases multiple HPV genotypes are detected [19,20,21]. The explanation of this phenomenon is, that mostly cytological samples are used to determine HPV genotypes, and such sources often contain transient or non-causal infections, that originated from the bulk population of exfoliated cervical cells and surface debris as well [22]. Considering these, the DNA from the FFPE cone tissue sample, as it rather originates from a pathologically confirmed lesion, is a more reliable source of HSIL-DNA to detect persistent infection. Thus, FFPE- HPV profile should be taken as the gold standard [22,23,24,25]. Hence, when applying cytological samples in clinical practice during the post-conization follow-up, detection of the same HPV genotypes, that was also presented in the cone specimen, could help to identify patients at higher risk of disease progression [26,27].

Data comparing HPV genotype profile of cytological and cervical FFPE cone samples at the time of diagnosis, from the same patient, are limited and incomplete in the literature and completely absent in Central-Eastern Europe.

Therefore, in a retrospective study, we aimed to determine the occurrence and agreement of HPV genotypes in women diagnosed with HSIL, by comparing the HPV genotype profile of cytological samples and cervical cone samples, applying a method which is sensitive to 35 different genotypes of HPV (18HR and 17LR). We think that our data could present additional evidence on the role of persistent HR-HPV infection in the development of HSIL or invasive cervical cancer. Moreover, by detecting a high number of HPV genotypes, our study allows us to draw attention to the relevance of HPV vaccines, especially to the widely used nonavalent vaccine (Gardasil 9; Merck), and to the occurrence of non-nonavalent vaccinated type (VT) HPVs, providing important information for vaccine development.

## 2. Materials and Methods

Archived conventional PAP smears and FFPE cone samples of 49 women with a diagnosis of HSIL were included in our retrospective study. The study was based on women that attended the Department of Obstetrics and Gynaecology and other outpatient clinics in Pécs, between 2019 and 2020, due to routine gynecological screening and diagnostic follow-up. Conization was performed within a 6 month period after cytological sampling in all cases and the genotyping was accomplished during the spring of 2021.

### 2.1. Sampling, Cytological and Histological Diagnoses

Cytological conventional PAP smears of each case were evaluated by three independent cytopathologists based on the Bethesda 2014 system. In the histological specimens, evaluation of the individual areas that mostly represent HSIL was made according to standard criteria, 2020 WHO classification, on hematoxylin and eosin-stained sections, observed by experienced histopathologists. For further processing, 3 series of 10 µm-thick slices of archived FFPE cervix cone samples were cut. Besides, exfoliated cervical cells from archived PAP smears were scraped by sterile scalpel and were suspended in 500 µL sterile gynecological (GYN) specimen preservation solution (CelltraZone) and were used for HPV genotype analysis.

### 2.2. Sample Preparation, HPV Detection, and Genotyping by HPV Direct Flow CHIP

HPV genotype determination was done by the HPV Direct Flow CHIP system (Master Diagnostica, Granada, Spain) at both sample materials. The test is able to differentiate between 18 high-risk and 17 low-risk HPV genotypes as, HR HPVs 16, 18, 26, 31, 33, 35, 39, 45, 51, 52, 53, 56, 58, 59, 66, 68, 73, and 82 and LR HPVs 6, 11, 40, 42, 43, 44, 54, 55, 61, 62, 67, 69, 70, 71, 72, 81, and 84. The system includes GP5+/GP6+-based PCR, reverse dot blot hybridization, and automatic readout. An additional fragment (268 bp) of the human beta-globin gene is co-amplified during the PCR to assure the quality of the input starting material. Samples were processed based on the manufacturer’s instruction following the protocol for cytology in GYN transport medium and paraffin-embedded tissue sections for direct PCR.

Processing of cytological samples: 500 µL of cytological samples were centrifuged for 2 min at 2000 rpm at room temperature. The pellets were dissolved in 400 µL PCR sterile water and the centrifugation step was repeated. The pellets were dissolved in 300 µL sterile water; 30 µL of the solution was used for PCR amplification.

Processing of FFPE tissue samples: Three 10 μm thick sections were dissolved in 400 μL mineral oil and then incubated at 95 °C for 2 min. After removing the mineral oil, 60 μL lysis buffer with 1.5 μL DNA release (Master Diagnostica, Granada, Spain) was added to the samples and incubated at 60 °C for 30 min, followed by inactivation at 98 °C for 10 min. Furthermore, 3 μL of crude cell extract supplemented with 27 μL DNase/RNase free water was used for PCR amplification. The lyophilized PCR HPV mix contained PCR buffer, dNTP(U/T), DNase/RNase free water, biotinylated primers, DNA polymerase, and UNG. The primers used are specific for the amplification of a fragment of the L1 region of the HPV genome. Besides, primers for the amplification of the human beta-globin gene are included and used as an internal control for the PCR reaction. The amplification cycling conditions in IANLONG PCR Thermal Cycler (Genesy 96T) were the following: pre-incubation at 25 °C for 10 min then 94 °C for 3 min; 15 cycles of denaturation at 94 °C for 30 s, annealing at 47 °C for 30 s and elongation at 72 °C for 30 s; 35 cycles of denaturation at 94 °C for 30 s, annealing at 65 °C for 30 s and elongation at 72 °C for 30 s and final elongation at 72 °C for 5 min; followed by cooling at 8 °C. Amplicons were denatured at 95 °C for 10 min then cooled on ice for 5 min.

The hybridization process was performed semi-automatically in hybriSpot (HS12) following the manufacturer’s instructions. The management of the samples, the images, the analysis, and the report of the results was performed by hybriSoft software.

### 2.3. Statistics

To compare PAP smears and FFPE cones of the patients we applied McNemar and McNemar–Bowker tests as appropriate. To observe agreement between the sample types (e.g., PAP and FFPE) we used weighted kappa statistics with a 95% confidence interval. In the case of genotype analysis, we calculated the ratios’ exact confidence interval, using the Poisson distribution and Poisson test. Only *p* values < 0.05 were considered to be statistically significant.

## 3. Results

### 3.1. Patients Characteristics

Overall, 49 women with a median age of 39 years (ranging from 17 to 64 years of age) were included in our retrospective study. The patients mostly resided in the southwest part of Hungary. All patients had high-grade cytology and histopathology. The HPV test was performed on conventional PAP smears and FFPE cone samples. The test, provided positive β-globin results in all cases, suggesting that the samples’ DNA quality and quantity were suitable for the analysis. All patients proved to be HPV positive.

### 3.2. HPV Prevalence and Agreement between the Relating Sample Types

**Figure 1:**
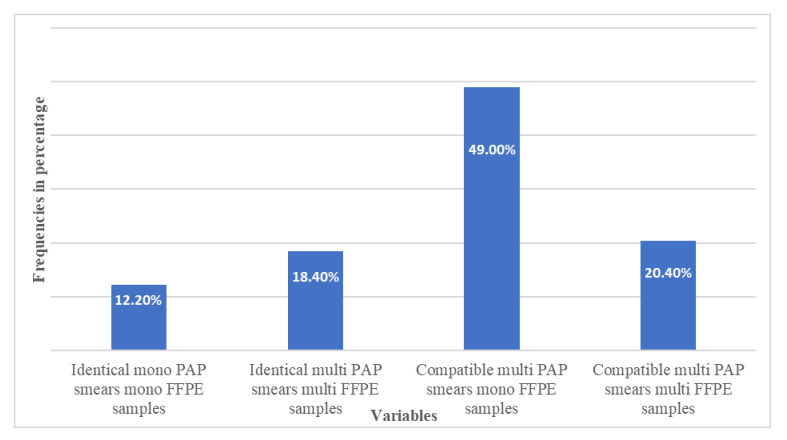
PAP smear and FFPE cone samples originated from the same patient in all 49 cases. Variables are based on the agreement of the human papillomavirus genotype prevalence in the two sample types. Most of the samples (49.00%) belong to the compatible category with multi-HPV infections in PAP smears coupled with mono-HPV infection in FFPE cone samples. Identical: the detected HPV genotypes are exactly the same in the PAP smears and their FFPE cone sample pairs. Compatible: at least one of the genotypes detected in multi-PAP smears was also found in their FFPE cone sample pairs.

HPV genotype classification was based on the HPV Direct Flow Chip Test’s (Master Diagnostica, Granada, Spain) description. HPV DNA was detected, in all of the 49 cases in both sample types. The PAP smears and FFPE samples showed identical genotype (HPV genotypes are the same in both sample types) in 15 (30.61%), compatible genotype (at least one of the genotypes detected in PAP smears also found in FFPE tissue samples) in 34 (69.38%) specimens, while discrepant genotype (present only in FFPE sample) was not found. It means that if we considered FFPE to reference genotype profile, the identity is 100% with the appropriate PAP smears. By examination of the agreement between the cytological and histological profiles, we found a moderate correlation (kappa value: 0.616 CI: 0.540–0.692), which is due to the fact that the sampling conditions are completely different. Figure 1 presents a detailed description of variables and case numbers in percentage. It indicates that most of the cases belong to the compatible category, with multi-infected PAP smear linking to mono-infected FFPE cone sample pair.

Of the 49 PAP smears, 6 (12.24%) appeared with mono-infection, while 43 (87.76%) occurred with multi-infections. In contrast, 30 (61.22%) of the 49 FFPE samples showed mono-infection, while 19 (38.78%) had a multi-HPV infection. By comparing the two categories we found that FFPE samples harbored a significantly higher number of mono-infection than PAP smears, *p* < 0.001.

When the infection numbers in multi-infected PAP smears and FFPE cone samples were compared, although the results were not significant due to the small sample size (*p* = 0.073), we found that the PAP smears harbored a higher number of HPV infections per sample than the FFPE cones, 3.21 (CI: 2.69–3.79) compared to 2.47 (CI: 1.82–3.29), respectively.

High-risk genotypes alone or combined with other high or low-risk genotypes were found in all PAP smears and FFPE tissue samples. Apart from the sample material, low-risk genotypes occurred as multi-infections only; of the 49 patients, the prevalence of low-risk infection was significantly lower in FFPE cone samples than in PAP smears, *p* = 0.001; 5 cases (10.20% CI: 3.31–23.81%) compared to 17 cases (34.69% CI: 20.21–55.55%) (Table 1).

### 3.3. Occurrence of the Specific Genotypes

From the 35 tested (18 HR and 17 LR), 16 high-risk and 12 low-risk HPV genotypes were detected in PAP smears, yielding a total number of 144 infections within the 49 samples.

In the FFPE cone samples, 16 high-risk and 6 low-risk HPV genotypes were found resulting in a total number of 77 infections. Supplementary Table S1. demonstrates the distribution of detected HPV genotypes in all the cases and both sample types. The three most frequently detected HPV genotypes in both materials were the nonavalent VT HPV 16 (18.75% and 29.87%), HPV 31 (10.41% and 11.68%), and the non-nonavalent VT HPV 51 (9.02% and 7.79%) in PAP smears and FFPE samples, respectively. The nonavalent VT HPV 33 occurred at the same frequency (7.79%) as the HPV 51 in the FFPE cones (Table 1).

In the PAP smears, three of the 35 HPV genotypes, namely, the nonavalent VT 16, 52, and 45 appeared as mono-infections. In contrast, in the FFPE cone samples, nine of the 35 HPV genotypes, the nonavalent VT 16, 31, 33, 45, 52, and 58 and the non-nonavalent VT 35, 68, and 82 appeared as mono-infections (Table 1). The significant result (*p* = 0.031) suggests that the number of HPV mono-infections in FFPE samples is higher than in PAP smears.

Regarding the occurrence of the nonavalent and non-nonavalent VT HPVs in PAP smears, 71 (49.30%) of the 144 infections were caused by the former types, while 73 (50.69%) were due to the latter ones. In addition, of the 73 non-vaccinated HPV infections, 52 (71.23%) were caused by high-risk HPVs, such as: HPV 51, 56, 35, 66, 53, 39, 73, 82, and 68. In the case of the FFPE samples, we found that 47 of all 77 infections (61.03%) were caused by nonavalent VT HPVs, while 30 (38.96%) belonged to the non-nonavalent VT HPVs; of which 26 (86.66%) were high-risk HPVs, such as HPV 51, 35, 53, 56, 82, 68, 73, 39, and 66. We also analyzed the agreements between the two samples from the same individual which are summarized in Supplementary Table S2, where the kappa values are also indicated. In the case of vaccinated type HPV (100% agreement), 19 of the 49 samples (38.78%) were identical, and in non-vaccinated type HPV it was only 8 of 49 samples (16.33%). Based on the results, a relatively large agreement is observed for vaccinated type HPV (κ = 0.678 (0.579–0.776)), while it was slightly smaller for non-vaccinated type HPV samples (κ = 0.500 (0.378–0.622)).

The most abundant genotype, the HPV 16, was found in 46.93% of the FFPE samples and 55.10% of PAP smears. In the case of PAP smears, the HPV 16 appeared significantly more frequently in the form of a multi-infection than in FFPE samples, where it mostly appeared as mono-infection, *p* = 0.002.

## 4. Discussion

Cervical conization is an effective treatment for HSIL, however, this treatment cannot eradicate high-risk HPV; a wide range of recurrence rates of HSIL after conization (0.35–69%) was reported, hence close follow-up of the patients is crucial [26,28,29]. Unfortunately, there is no consensus regarding the optimal follow-up and frequency of control examinations of patients in the post-treatment period. [30] Regular control examinations, probably for an extended period, may also be reasonable because of the increased risk of other HPV-related cancer types in those females treated with HSIL. [31] Data on persistent HPV distribution in HSIL patients is essential for understanding the formation of HPV-associated cervical lesions. FFPE tissue specimens, selected through microscopic examination, are thought to be the most reliable source to determine persistent HPV DNA. However, while formalin fixation often causes technical difficulties, such as low DNA recovery rate, fragmentation, cross-linking, etc., the preferred method to determine HPV genotype profile both at the time of diagnosis and during the post-conization follow up, is generally based on exfoliated cervical cells, even though such source contains many transient episomal HPV forms as well. During the post-conization follow-up, focusing on type-specific clearance is important in the consideration of treatment efficiency; along with decision making for post excision treatment with additional considerations for repeated excision in the event of positive margins.

In our retrospective study, we compared and analyzed the agreement of the HPV genotype profile of PAP smears and FFPE cone samples, originated within six months, from the same 49 women diagnosed with HSIL. The sensitivity and specificity of the applied method (HPV Direct Flow Chip) had been proved in independent studies [32,33,34,35]; in addition, in the current study the test appeared to be suitable for HPV genotyping both from conventional PAP smears and FFPE tissue specimens; 100% of the samples gave reliable data for the analysis.

The overall agreement between PAP smears and FFPE cone samples was very good (100%), suggesting that cytological samples are reliable sources for verifying the presence of certain HPV genotypes.

Either in the form of mono- or multi-infection, our results underlined the importance of high-risk HPV genotypes in the formation of precancerous HSIL, as all of the 49 PAP and FFPE samples harbored high-risk HPV genotypes.

Cervical coinfection with more than one HPV genotype is common; women infected with one HPV type are more likely to harbor additional genotypes [36,37]. Indeed, many studies suggest that multiple HPV infections bear a greater risk in either the development, progression, or both, of lesions [38]. To date, there is no consensus whether multiple HPV types occur randomly or through competitive or cooperative relationships. Some studies suggest that multiple HPV types can act synergistically in cervical carcinogenesis, while others favor rather random patterns of different HPV types [37,39,40]. Although our study has not been extended to linkage analysis between the different HPV genotypes, an interesting finding of our work is the significantly higher (*p* < 0.001) proportion of multiple infections detected in the 49 PAP smears (87.76%) compared to the FFPE cone samples (38.78%).

HPV genotypes integrate into the human cell/genome, achieving increased viral oncoprotein expression and increased mRNA stability, which generates the imbalance of cellular genes that leads to carcinogenesis. In rare cases, multiple viral genomes could integrate, however, most of these become epigenetically silenced [41,42,43,44,45]. Though 38.78% of the FFPE samples occurred with multi-infections when comparing the infection number per sample, we found that although results are not significant, a multi-infected PAP smear harbors more HPV types than a multi-infected FFPE sample, 3.21 compared to 2.47, respectively, suggesting that even in case of multi-infections, PAP smears contain more transient and non-causal HPV infection than FFPE tissue samples do. A possible explanation of multi-infected FFPE cone samples is that routine pathological diagnosis of squamous intraepithelial lesions is based on the highest grade of SIL detected in the whole biopsy. This conceals the underlying architectural complexity of many SILs with areas of different grades and HPV infections [19]. However, assessment of the initial HPV profile of the tissue sample has substantial information regarding patient follow-up.

Apart from the sample material, the most abundant genotype was the nonavalent VT HPV 16, with 46.93% and 55.10% of FFPE and PAP samples, respectively, followed by the nonavalent VT HPV 31, and non-nonavalent 51, together with the nonavalent VT 33 in the FFPE cone samples. These findings, together with the relatively low prevalence of nonavalent VT HPV 18, are consistent with a previous report in Hungary, although that study only examined cervical swab samples [46].

Indeed, using the same method on two types of samples that originated from the same patients, we were able to analyze the distribution of 35 different HPV types giving reliable information about their infection forms (multi- or mono-infection), and thereby their oncogenic potential in cervical cancerogenesis. In FFPE cone samples the nonavalent VT HPVs 16, 31, 33, 45, and 52 and non-nonavalent VT HPVs 35, 68, and 82 appeared as mono-infections.

Today the most effective weapons against HPV infection are vaccines. Currently, three effective HPV prophylactic vaccines are available, all of which contain HPV L1 proteins that are capable of forming virus-like particles (VLP). Of these, the nonavalent Gardasil 9 (Merck) covers the most HPV types, such as HPVs 6, 11, 16, 18, 31, 33, 45, 52, and 59. This vaccine was approved by the FDA in late 2014; in Hungary, it is available since 2015 [35,47]. Numerous studies focus on the cross-protection capability of these vaccines, but the results are still confusing [35,47,48]. Preliminary data indicate that HPV vaccination with 2 or 4-valent anti-HPV VLP vaccines significantly reduced the HPV 16, 18, and 31 DNA positivity rates in LBC samples of patients who previously tested positive [49]. Moreover, according to a recent meta-analysis, HPV vaccination in adjuvant settings was also able to reduce the recurrence rate of cervical intraepithelial neoplasia [50]. These recent data suggest the potential benefit of different vaccines on cervical pathology of HPV-infected patients, however larger cohort studies are needed to define the real value of the individual vaccines.

An essential finding of our study was the high rate of non-nonavalent VT HPV prevalence in both sample types—50.69% in PAP smears and 38.96% in FFPE cone specimens from all infections (144 and 77, respectively). The results of this study suggest that either single or multiple nonavalent VT, as well as non-nonavalent VT HPVs, are associated with HSIL.

## 5. Conclusions

Reports on HPV genotype profile and agreement of cytological and cervical FFPE cone samples with HSIL are rare and up to now, not available from Central-Eastern Europe. The ultimate goal of our study was to draw attention to the importance of HPV genotype profiling of cone samples in precancerous HSILs. Cone samples are the references regarding the diagnosis and the assessment of therapy efficiency during post-conization follow-up. Our results confirmed strong concordance between HPV genotypes found in cytological and tissue samples, suggesting that cytological samples are reliable sources for verifying the prevalence of certain, oncogenic HPV genotypes present in cone specimens.

By applying detailed HPV genotype analysis, we pointed out the presence of non-vaccinated HPVs as well in HSIL cases; in addition, 71.23% and 86.66% of such infections were caused by high-risk non-vaccinated HPV genotypes in PAP smears and FFPE cone samples, respectively. Together with possible interactions between different HPV types, these findings should be considered in the design of vaccination strategies and highlight the need for the development of wider-spectrum vaccines against HPV.

## Figures and Tables

**Table 1:** Prevalence of human papillomavirus (HPV) genotypes and infection numbers in FFPE cone samples and PAP smears among patients diagnosed with high-grade squamous intraepithelial lesions (HSIL). VT: nonavalent vaccinated type HPV; NVT: non-nonavalent vaccinated type HPV.

Number of Infections (%)						
HPV Genotypes	Number of Mono-Infection		Number of Multi-Infection		Number of Total Infections	
High-Risk (HR) HPV Genotypes	FFPE Cone Samples (*n* = 30)	PAP Smears (*n* = 6)	FFPE Cone Samples (*n* = 47)	PAP Smears (*n* = 138)	FFPE Cone Samples (*n* = 77)	PAP Smears (*n* = 144)
16 (VT)	14 (46.66%)	4 (66.66%)	9 (19.14%)	23 (16.66%)	23 (29.87%)	27 (18.75%)
31 (VT)	6 (20.00%)	-	3 (6.38%)	15 (10.86%)	9 (11.68%)	15 (10.41%)
51 (VT)	-	-	6 (12.76%)	13 (9.42%)	6 (7.79%)	13 (9.02%)
33 (VT)	4 (13.33%)	-	2 (4.25%)	9 (6.52%)	6 (7.79%)	9 (6.25%)
35 (NVT)	1 (3.33%)	-	4 (8.51%)	7 (5.07%)	5 (6.49%)	7 (4.86%)
18 (VT)	-	-	3 (6.38%)	8 (5.79%)	3 (3.89%)	8 (5.55%)
52 (VT)	1 (3.33%)	1 (16.66%)	2 (4.25%)	4 (2.89%)	3 (3.89%)	5 (3.47%)
53 (NVT)	-	-	3 (6.38%)	4 (2.89%)	3 (3.89%)	4 (2.77%)
56 (NVT)	-	-	3 (6.38%)	7 (5.07%)	3 (3.89%)	7 (4.86%)
58 (VT)	1 (3.33%)	-	2 (4.25%)	10 (7.24%)	3 (3.89%)	10 (6.94%)
45 (VT)	1 (3.33%)	1 (16.66%)	1 (2.12%)	3 (2.17%)	2 (2.59%)	4 (2.77%)
82 (NVT)	1 (3.33%)	-	1 (2.12%)	2 (1.44%)	2 (2.59%)	2 (1.38%)
68 (NVT)	1 (3.33%)	-	-	1 (0.72%)	1 (1.29%)	1 (0.69%)
73 (NVT)	-	-	1 (2.12%)	2 (1.44%)	1 (1.29%)	2 (1.38%)
39 (NVT)	-	-	1 (2.12%)	2 (1.44%)	1 (1.29%)	2 (1.38%)
66 (NVT)	-	-	1 (2.12%)	4 (2.89%)	1 (1.29%)	4 (2.77%)
**Low-risk (LR) HPV genotypes**						
42 (NVT)	-	-	1 (2.12%)	4 (2.89%)	1 (1.29%)	4 (2.77%)
11 (VT)	-	-	1 (2.12%)	-	1 (1.29%)	-
70 (NVT)	-	-	1 (2.12%)	3 (2.17%)	1 (1.29%)	3 (2.08%)
54 (NVT)	-	-	1 (2.12%)	2 (1.44%)	1 (1.29%)	2 (1.38%)
62/81 (NVT)	-	-	1 (2.12%)	5 (3.62%)	1 (1.29%)	5 (3.47%)
6 (VT)	-	-	-	3 (2.17%)	-	3 (2.08%)
67 (NVT)	-	-	-	3 (2.17%)	-	3 (2.08%)
44/55 (NVT)	-	-	-	1 (0.72%)	-	1 (0.69%)
84 (NVT)	-	-	-	1 (0.72%)	-	1 (0.69%)
61 (NVT)	-	-	-	1 (0.72%)	-	1 (0.69%)
43 (NVT)	-	-	-	1 (0.72%)	-	1 (0.69%)

## Data Availability

Data are available in Supplementary Tables S1 and S2.

## Supplementary Materials

The following supporting information can be downloaded at: https://www.mdpi.com/article/10.3390/vaccines10050748/s1. Table S1: Distribution of HPV genotypes in each sample; Table S2: Numbers of agreements and kappa values

## Abbreviations

CI	confidence interval
FFPE	formalin-fixed paraffin-embedded
GYN	gynecological
HPV	human papillomavirus
HSIL	high-grade squamous intraepithelial lesions
HR	high-risk
LR	low-risk
LSIL	low-grade squamous intraepithelial lesions
PAP	Papanicolaou
SIL	squamous intraepithelial lesions
VT	vaccinated type

## References

[1] Graham S.V. (2017). The human papillomavirus replication cycle, and its links to cancer progression: A comprehensive review. Clin. Sci..

[2] (2012). Papillomavirus Episteme: A Comprehensive Papillomaviridae Database and Analysis Resource. https://pave.niaid.nih.gov/#home.

[3] Van Doorslaer K., Li Z., Xirasagar S., Maes P., Kaminsky D., Liou D., Sun Q., Kaur R., Huyen Y., McBride A.A. (2017). The Papillomavirus Episteme: A major update to the papillomavirus sequence database. Nucleic Acids Res..

[4] WHO, International Agency for Research on Cancer (2011). IARC Monographs on the Evaluation of Carcinogenic Risks to Humans.

[5] Smith J.S., Lindsay L., Hoots B., Keys J., Franceschi S., Winer R., Clifford G.M. (2007). Human papillomavirus type distribution in invasive cervical cancer and high-grade cervical lesions: A meta-analysis update. Int. J. Cancer.

[6] Clifford G.M., Smith J.S., Aguado T., Franceschi S. (2003). Comparison of HPV type distribution in high-grade cervical lesions and cervical cancer: A meta-analysis. Br. J. Cancer.

[7] Clifford G.M., Smith J.S., Plummer M., Muñoz N., Franceschi S. (2003). Human papillomavirus types in invasive cervical cancer worldwide: A meta-analysis. Br. J. Cancer.

[8] Tachez R., Smahelova J., Saláková M., Arbyn M., Rob L., Skapa P., Jirásek T., Hamsikova E. (2011). Human Papillomavirus Genotype Distribution in Czech Woman an Men with Diseases Etiologycally Linked to HPV. PLoS ONE.

[9] Man I., Vänskä S., Lehtinen M., Bogaards J. (2021). Human Papillomavirus Genotype Replacement: Still Too Early to Tell?. J. Infect. Dis..

[10] Sousa H., Tavares A., Campos C., Marinho-Dias J., Brito M., Medeiros R., Baldaque I., Lobo C., Leça L., Monteiro P. (2019). High-Risk human papillomavirus genotype distribution in the Northern region of Portugal: Data from regional cervical cancer screening program. Papillomavirus Res..

[11] Stanley M. (2010). Pathology and epidemiology of HPV infection in females. Gynecol. Oncol..

[12] De Brot L., Pellegrini B., Moretti S.T., Carraro D.M., Soares F.A., Rocha R.M., Baiocchi G., Werneck da Cunha I., Piana de Andrade V. (2017). Infections with Multiple High-Risk HPV Types Are Associated With High-Grade and Persistent Low-Grade Intraepithelial Lesions of the Cervix. Cancer Cytopathol..

[13] Gheit T. (2019). Mucosal and Cutaneous Human Papillomavirus Infections and Cancer Biology. Front. Oncol..

[14] Schiffman M., Castel P.E., Jeronimo J., Rodriguez A.C., Wacholder S. (2007). Human papillomavirus and cervical cancer. Lancet.

[15] Maucort-Boulch D., Plummer M., Castle P.E., Demuth F., Safaeian M., Wheeler C.M., Schiffman M. (2010). Predictors of human papillomavirus persistence among women with equivocal or mildly abnormal cytology. Int. J. Cancer.

[16] Cubie H.A. (2013). Diseases associated with human papillomavirus infection. Virology.

[17] Veldhuijzen N.J., Snijders P.J.F., Reiss P., Meijer C.J.L.M., van de Wijgert J.H.H.M. (2010). Factors affecting transmission of mucosal human papillomavirus. Lancet Infect. Dis..

[18] Zur Hausen H. (2009). Papillomaviruses in the causation of human cancers—A brief historical account. Virology.

[19] Quint W., Jenkins D., Molijn A., Struijk L., van de Sandt M., Doorbar J., Mols J., Van Hoof C., Hardt K., Struyf F. (2012). One virus, one lesion--individual components of CIN lesions contain a specific HPV type. J. Pathol..

[20] Plummer M., Vaccarella S., Franceschi S. (2011). Multiple Human Papillomavirus Infections: The Exception or the Rule?. J. Infect. Dis..

[21] Li N., Franceschi S., Howell-Jones R., Snijders P.J., Clifford G.M. (2010). Human papillomavirus type distribution in 30,848 invasive cervical cancers worldwide: Variation by geographical region, histological type and year of publication. Int. J. Cancer.

[22] Torii Y., Fujii T., Kukimoto I., Saito M., Iwata T., Takahashi H., Ichikawa R., Kawai S., Otani S., Aoki D. (2016). Comparison of methods using paraffin-embedded tissues and exfoliated cervical cells to evaluate human papillomavirus genotype attribution. Cancer Sci..

[23] Serrano B., de Sanjosé S., Tous S., Quiros B., Muñoz N., Bosch X., Alemany L. (2015). Human papillomavirus genotype attribution for HPVs 6, 11, 16, 18, 31, 33, 45, 52 and 58 in female anogenital lesions. Eur. J. Cancer.

[24] Insinga R.P., Liaw K.L., Johnson L.G., Madeleine M.M. (2008). A systematic review of the prevalence and attribution of human papillomavirus types among cervical, vaginal, and vulvar precancers and cancers in the United States. Cancer Epidemiol. Biomarkers Prev..

[25] Kim G., Cho H., Lee D., Park S., Lee J., Wang H.Y., Kim S., Park K.H., Lee H. (2017). Comparison of FFPE histological versus LBP cytological samples for HPV detection and typing in cervical cancer. Exp. Mol. Pathol..

[26] Bottari F., Passerini R., Renne G., Guerrier M.E., Sandri M.T., Li A., Orlandini A., Iacobone A.D. (2021). Onclarity Performance in Human Papillomavirus DNA Detection in Formalin-Fixed Paraffin-Embedded Cervical Samples. J. Low. Genit. Tract Dis..

[27] Bonde J., Bottari F., Iacobone A.D., Cocuzza C.E., Sandri M.T., Bogliatto F., Khan K.S., Ejegod D.M., Gary D.S., Andrews J.C. (2021). Human Papillomavirus Same Genotype Persistence and Risk: A Systematic Review. J. Low. Genit. Tract Dis..

[28] Huang H.J., Tung H.J., Yang L.Y., Chao A., Tang Y.H., Chou H.H., Chang W.Y., Wu R.C., Huang C.C., Lin C.Y. (2021). Role of human papillomavirus status after conization for high-grade cervical intraepithelial neoplasia. Int. J. Cancer.

[29] Chao A., Lin C.T., Hsueh S., Chou H.H., Chang T.C., Chen M.Y., Lai C.H. (2004). Usefulness of human papillomavirus testing in the follow-up of patients with high-grade cervical intraepithelial neoplasia after conization. Am. J. Obstet. Gynecol..

[30] Kyrgiou M., Tsoumpou I., Vrekoussis T., Martin-Hirsch P., Arbyn M., Prendiville W., Mitrou S., Koliopoulos G., Dalkalitsis N., Stamatopoulos P. (2006). The up-to-date evidence on colposcopy practice and treatment of cervical intraepithelial neoplasia: The Cochrane colposcopy & cervical cytopathology collaborative group (C5 group) approach. Cancer Treat Rev..

[31] Kalliala I., Athanasiou A., Veroniki A.A., Salanti G., Efthimiou O., Raftis N., Bowden S., Paraskevaidi M., Aro K., Arbyn M. (2020). Incidence and mortality from cervical cancer and other malignancies after treatment of cervical intraepithelial neoplasia: A systematic review and meta-analysis of the literature. Ann Oncol..

[32] Herrarez-Hernandez E., Preda O., Alonso S., Pardo R.S., Olmo A. (2013). Detection and Genotyping of Human Papillomavirus DNA in Formalin-Fixed Pareffin-Embadded Specimens with the HPV Direct Flow CHIP System. Open Virol. J..

[33] Kocjan B.J., Hošnjak L., Poljak M. (2016). Detection of alpha human papillomaviruses in archival formalin-fixed, paraffin-embedded (FFPE) tissue specimens. J. Clin. Virol..

[34] Herrarez-Hernandez E., Alvarez-Perez M., Navarro-Bustos G., Esquivias J., Alonso S., Aneiros-Fernandez J., Lacruz-Pelea C., Sanchez-Aguera M., Santamaria J.S., Chanon de Antonio J. (2013). HPV Direct Flow CHIP: A new human papillomavirus genotyping method based on direct PCR from crude-cell extras. J. Virol. Methods.

[35] Rideg O., Oszter A., Makk E., Kálmán E., Farkas K., Tornóczky T., Kovács K. (2021). Wide Spectrum Analysis of Human Papillomavirus Genotypes in External Anogenital Warts. Vaccines.

[36] Chaturvedi A.K., Myers L., Hammons A.F., Clark R.A., Dunlap K., Kissinger P.J., Hagensee M.E. (2005). Prevalence and clustering patterns of human papillomavirus genotypes in multiple infections. Cancer Epidemiol. Biomarkers Prev..

[37] Chaturvedi A.K., Katki H.A., Hildesheim A., Rodríguez A.C., Quint W., Schiffman M., Van Doorn L.J., Porras C., Wacholder S., Gonzalez P. (2011). Human papillomavirus infection with multiple types: Pattern of coinfection and risk of cervical disease. J. Infect. Dis..

[38] Trottier H., Mahmud S., Costa M.C., Sobrinho J.P., Duarte-Franco E., Rohan T.E., Ferenczy A., Villa L.L., Franco E.L. (2006). Human papillomavirus infections with multiple types and risk of cervical neoplasia. Cancer Epidemiol. Biomarkers Prev..

[39] Chagas B.S., Comar M., Gurgel A.P., Paiva S., Seraceni S., de Freitas A.C., Crovella S. (2015). Association Study between Cervical Lesions and Single or Multiple Vaccine-Target and Non-Vaccine Target Human Papillomavirus (HPV) Types in Women from Northeastern Brazil. PLoS ONE.

[40] Dickson E.L., Vogel R.I., Geller M.A., Downs L.S. (2014). Cervical cytology and multiple type HPV infection: A study of 8182 women ages 31-65. Gynecol. Oncol..

[41] Mejlhede N., Pedersen B.V., Frisch M., Fomsgaard A. (2010). Multiple human papilloma virus types in cervical infections: Competition or synergy?. APMIS.

[42] Salazar K.L., Zhou H.S., Xu J., Peterson L.E., Schwartz M.R., Mody D.R., Ge Y. (2015). Multiple Human Papilloma Virus Infections and Their Impact on the Development of High-Risk Cervical Lesions. Acta Cytol..

[43] Moody C.A., Laimins L.A. (2010). Human papillomavirus oncoproteins: Pathways to transformation. Nat. Rev. Cancer.

[44] Groves I.J., Coleman N. (2015). Pathogenesis of human papillomavirus-associated mucosal disease. J. Pathol..

[45] Kyo S., Klumpp D.J., Inoue M., Kanaya T., Laimins L.A. (1997). Expression of AP1 during cellular differentiation determines human papillomavirus E6/E7 expression in stratified epithelial cells. J. Gen. Virol..

[46] Galamb A., Pajor A., Langmár Z., Sobel G. (2011). Az első magyarországi humán papillomavírus központ tapasztalatai (2007–2011) [Results of the first human papilloma virus center in Hungary (2007–2011)]. Orv. Hetil..

[47] Nakagawa M., Greenfield W., Moerman-Herzog A., Coleman H.N. (2015). Cross-Reactivity, Epitope Spreading, and De Novo Immune Stimulation Are Possible Mechanisms of Cross-Protection of Nonvaccine Human Papillomavirus (HPV) Types in Recipients of HPV Therapeutic Vaccines. Clin. Vaccine Immunol..

[48] Li Z., Song S., He M., Wang D., Shi J., Liu X., Li Y., Chi X., Wei S., Yang Y. (2018). Rational design of a triple-type human papillomavirus vaccine by compromising viral-type specificity. Nat. Commun..

[49] Valasoulis G., Pouliakis A., Michail G., Kotarridi C., Spathis A., Kyrgiou M., Paraskevaidis E., Daponte A. (2020). Alterations of HPV-Related Biomarkers after Prophylactic HPV Vaccination. A Prospective Pilot Observational Study in Greek Women. Cancers.

[50] Di Donato V., Caruso G., Petrillo M., Kontopantelis E., Palaia I., Perniola G., Plotti F., Angioli R., Muzii L., Benedetti Panici P. (2021). Adjuvant HPV Vaccination to Prevent Recurrent Cervical Dysplasia after Surgical Treatment: A Meta-Analysis. Vaccines.

