# Supplementary material for: High Prevalence of Non-Vaccinated Oncogenic Human Papillomavirus Genotypes in High-Grade Squamous Intraepithelial Lesions of the Cervix: Thought-Provoking Results of a Detailed HPV Genotype Analysis
Source: Vaccines (Basel). 2022 May 10;10(5):748. doi: 10.3390/vaccines10050748 (PMC9146889; doi:10.3390/vaccines10050748)
Supplement: Supplementary file 1 [file vaccines-10-00748-s001.zip › vaccines-1673616-supplementary.pdf]

Supplementary Table S1.

Distribution of HPV genotypes in each sample. C: cytology, (means PAP smear); H: histology, (means FFPE sample) of the same patient. High-risk genotypes are highlighted in red colour, low-risk genotypes are in green colour. The „o" sign indicates the cytology for easy distinction.

HPV GENOTYPES

CASES		6	11	16	18	26	31	33	35	39	40	42	43	44/55	45	51	52	53	54	56	58	59	61	62/81	66	67	68	69	70	71	72	73	82	84
1	C				o										o					o	o													
	H																																	
2	C			o			o														o													
	H																																	
3	C				o										o																			
	H																																	
4	C								o														o	o	o							o		o
	H																																	
5	C			o	o							o								o														
	H																																	
6	C			o									o						o		o					o								
	H																																	
7	C			o														o																
	H																																	
8	C			o	o																o													
	H																																	
9	C			o																									o					
	H																																	
10	C				o		o														o													
	H																																	
11	C			o																														
	H																																	
12	C						o		o																									
	H																																	
13	C			o												o																o		
	H																																	
14	C				o										o	o		o			o	o							o				o	
	H																																	
15	C			o			o			o		o				o																		
	H																																	
16	C			o																														
	H																																	
17	C							o												o														
	H																																	
18	C																o																	
	H																																	
19	C	o							o								o																	
	H																																	
20	C			o				o	o																									
	H																																	
21	C						o		o					o												o								
	H																																	
22	C			o																														
	H																																	
23	C																								o				o					
	H																																	
24	C			o																o														
	H																																	
25	C						o									o					o													
	H																																	
26	C	o		o					o			o					o																	
	H																																	
27	C			o				o																o										
	H																																	
28	C			o																	o	o												
	H																																	
29	C			o																o														
	H																																	
30	C				o		o																											
	H																																	
31	C			o			o	o		o																	o							
	H																																	
32	C			o			o																											
	H																																	
33	C						o		o							o		o																
	H																																	
34	C															o		o						o										
	H																																	
35	C			o												o				o	o				o									
	H																																	
36	C			o			o					o				o								o										
	H																																	
37	C			o												o								o										
	H																																	
38	C			o				o								o																		
	H																																	
39	C						o	o																		o								
	H																																	
40	C						o	o								o									o									
	H																																	
41	C			o			o															o												
	H																																	
42	C							o													o													
	H																																	
43	C							o									o																	
	H																																	
44	C	o																															o	
	H																																	
45	C														o																			
	H																																	
46	C			o	o																													
	H																																	
47	C															o			o	o														
	H																																	
48	C			o			o									o	o																	
	H																																	
49	C			o																														
	H																																	

Supplementary Table S2.

Numbers of agreements and kappa values between PAP smears and FFPE cone samples in the cases of vaccinated and non-vaccinated virus types. n.a.: kappa value is not applicable.

	**Vaccinated type of HPV**					**Non-vaccinated type of HPV**				
	**PAP / FFPE**				**Kappa**	**PAP / FFPE**				**Kappa**
**case**	+/+	+/-	-/+	-/-		+/+	+/-	-/+	-/-	
1	1	2	0	4	0.364	0	1	0	25	n.a.
2	1	2	0	4	0.364	0	0	0	26	n.a.
3	2	0	0	5	1.000	0	0	0	26	n.a.
4	0	1	0	6	n.a.	1	5	0	20	0.235
5	2	0	0	5	1.000	1	1	1	23	0.458
6	2	0	0	5	1.000	0	3	1	22	-0.061
7	1	0	0	6	1.000	1	0	0	25	1.000
8	1	2	0	4	0.364	0	0	0	26	n.a.
9	1	0	0	6	1.000	0	1	0	25	n.a.
10	3	0	0	4	1.000	0	0	0	26	n.a.
11	1	0	0	6	1.000	0	0	0	26	n.a.
12	1	0	0	6	1.000	1	0	0	25	1.000
13	0	1	0	6	n.a.	2	0	0	24	1.000
14	0	3	0	4	n.a.	3	3	0	20	0.606
15	1	1	0	5	0.588	2	1	0	23	0.780
16	1	0	0	6	1.000	0	0	0	26	n.a.
17	1	0	1	5	0.588	0	1	0	25	n.a.
18	1	0	0	6	1.000	0	0	0	26	n.a.
19	1	0	0	6	1.000	1	1	0	24	0.649
20	1	1	0	5	0.588	0	1	0	25	n.a.
21	1	0	0	6	1.000	0	3	0	23	n.a.
22	1	0	0	6	1.000	0	0	0	26	n.a.
23	0	0	0	7	n.a.	2	0	0	24	1.000
24	1	0	0	6	1.000	1	0	0	25	1.000
25	1	1	0	5	0.588	0	1	0	25	n.a.
26	1	1	0	5	0.588	0	3	0	23	n.a.
27	1	1	0	5	0.588	0	1	0	25	n.a.
28	1	1	0	5	0.588	0	1	0	25	n.a.
29	1	0	0	6	1.000	1	0	0	25	1.000
30	1	1	0	5	0.588	0	1	0	25	n.a.
31	0	3	0	4	n.a.	0	0	1	25	n.a.
32	1	1	1	4	0.300	0	0	0	26	n.a.
33	1	0	0	6	1.000	3	0	0	23	1.000
34	0	0	0	7	n.a.	2	1	0	23	0.780

35	1	1	0	5	0.588	0	3	0	23	n.a.
36	1	1	0	5	0.588	0	3	0	23	n.a.
37	1	0	0	6	1.000	0	2	0	24	n.a.
38	1	1	0	5	0.588	0	1	0	25	n.a.
39	1	1	0	5	0.588	0	1	0	25	n.a.
40	1	1	0	5	0.588	0	2	0	24	n.a.
41	1	1	0	5	0.588	0	1	0	25	n.a.
42	1	1	0	5	0.588	0	0	0	26	n.a.
43	1	1	0	5	0.588	0	0	0	26	n.a.
44	0	0	0	7	n.a.	1	1	0	24	0.649
45	1	0	0	6	1.000	0	0	0	26	n.a.
46	1	1	0	5	0.588	0	0	0	26	n.a.
47	0	0	0	7	n.a.	3	0	0	23	1.000
48	2	1	0	4	0.696	0	1	0	25	n.a.
49	1	0	0	6	1.000	0	0	0	26	n.a.
